# Neoadjuvant almonertinib as a bridging strategy to surgery in resectable lung cancer with active COVID-19: a case report

**DOI:** 10.3389/fphar.2026.1808763

**Published:** 2026-04-28

**Authors:** Shihui Sun, Henghui Bian, Lili Jin, Jianbin Zhang

**Affiliations:** 1 Department of Thoracic Surgery, Huzhou Central Hospital, Affiliated Central Hospital of Huzhou University, Huzhou, Zhejiang, China; 2 Department of Thoracic Surgery, Huzhou Central Hospital, The Fifth School of Clinical Medicine, Zhejiang Chinese Medical University, Huzhou, Zhejiang, China; 3 Department of Central Laboratory, Huzhou Central Hospital, The Fifth School of Clinical Medicine, Zhejiang Chinese Medical University, Huzhou, Zhejiang, China

**Keywords:** almonertinib, bridging therapy, case report, COVID-19, neoadjuvant therapy, non-small cell lung cancer

## Abstract

While surgical postponement is recommended for patients with resectable non-small cell lung cancer (NSCLC) and active COVID-19, safe and effective bridging therapies during the delay remain poorly defined, especially for epidermal growth factor receptor (EGFR)-mutant disease. We report a 71-year-old woman with resectable EGFR-mutant lung adenocarcinoma and concurrent active COVID-19 pneumonia. After multidisciplinary consensus, neoadjuvant almonertinib (110 mg daily) was administered as a bridging strategy. After 10 weeks, imaging showed significant tumor regression and complete resolution of pulmonary infiltrates. The patient subsequently underwent successful thoracoscopic radical resection. Pathological examination revealed less than 5% viable tumor cells, meeting criteria for major pathologic response (MPR). Adjuvant almonertinib was started on postoperative day 2. At 36-month follow-up, she remains disease-free with no significant treatment-related adverse events observed. This case demonstrates a potentially viable approach that warrants further investigation in this high-risk clinical scenario, offering a management template that simultaneously addresses infection resolution and tumor control to enable curative surgery.

## Introduction

The concurrent presentation of resectable EGFR-mutant NSCLC and acute SARS-CoV-2 infection poses a challenging clinical scenario. International consensus guidelines, informed by large-scale cohort studies, strongly advise postponing elective cancer surgery during active COVID-19 to mitigate the substantially increased perioperative morbidity and mortality (COVIDSurg Collaborative, 2020; COVIDSurg, 2021). However, for patients with locally advanced, potentially curable disease, this mandated delay carries a tangible risk of tumor progression, which may ultimately compromise resectability and long-term survival.

This clinical dilemma underscores the need to establish an effective bridging strategy during the surgical delay. Current neoadjuvant standards for locally advanced NSCLC encompass immune checkpoint inhibitor (ICI) therapy and platinum-based chemotherapy. Although neoadjuvant immunotherapy combined with chemotherapy has demonstrated substantial efficacy, as evidenced by the phase III CheckMate 816 trial (Forde et al., 2022), its utility is markedly limited for EGFR-mutant NSCLC—a subgroup characterized by inferior responses to ICIs (Gainor et al., 2016)—and its associated risk of immune-related pneumonitis warrants caution during active COVID-19 (Luo et al., 2020). Similarly, platinum-doublet chemotherapy has also been established as an effective neoadjuvant approach (NSCLC Meta-analysis Collaborative Group, 2014), but its myelosuppressive effects raise significant concern in the context of concurrent SARS-CoV-2 infection (Kuderer et al., 2020).

These limitations highlight the need for a targeted strategy that delivers potent antitumor activity without compounding immunosuppression or pulmonary toxicity. Third-generation EGFR tyrosine kinase inhibitors (TKIs), characterized by high target specificity and superior systemic tolerability, represent a compelling theoretical candidate (Ramalingam et al., 2020). They can deliver significant antitumor activity without the immunosuppressive effects of chemotherapy or the pneumonitis risk associated with ICIs. However, their use as a bridging strategy prior to surgery in patients with EGFR-mutant NSCLC and active COVID-19 has not been reported.

In this article, we present a case in which neoadjuvant almonertinib was successfully employed as a novel bridging strategy in a patient with resectable EGFR-mutant NSCLC and active COVID-19. This strategy safely bridged the period of active infection, achieved substantial tumor downstaging, and ultimately enabled curative surgery.

## Case description

**FIGURE 1:**
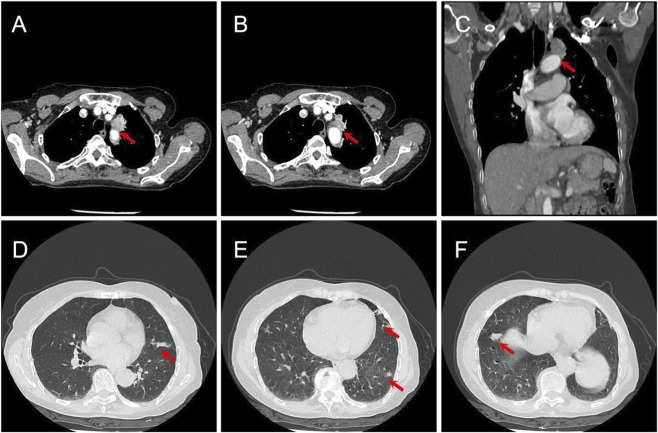
Baseline imaging findings at diagnosis. **(A–C)** Contrast-enhanced chest CT images show the primary left upper lobe tumor (arrows) with an indistinct border and loss of the fat plane adjacent to the mediastinum, consistent with clinical T4 (cT4) staging due to suspected mediastinal pleural invasion. **(D–F)** Multiple exudative lesions in both lungs, radiographically confirmed as COVID-19 pneumonia.

A 71-year-old never-smoking woman was referred to our institution in January 2023 during the COVID-19 pandemic. Her ECOG performance status was 1, and she had no history of hypertension, diabetes, cardiovascular disease, or chronic lung disease. A chest computed tomography (CT) scan performed for symptomatic COVID-19 pneumonia management incidentally revealed a 29-mm subpleural mass in the left upper lobe. Her medical history was unremarkable. At presentation, she reported a cough with expectoration but denied fever, chest pain, hemoptysis, or dyspnea. Physical examination showed stable vital signs (temperature 36.5 °C, heart rate 78 bpm, respiratory rate 18 bpm, blood pressure 125/80 mmHg, and oxygen saturation 97% on room air) and clear breath sounds without rales or rhonchi. Laboratory tests, including white blood cell count, C-reactive protein, and procalcitonin, were within normal limits. Serum carcinoembryonic antigen (CEA) was elevated at 7.79 ng/mL (normal range: 0–5.00 ng/mL). Pulmonary function tests revealed moderate mixed ventilatory dysfunction (FEV1: 1.42 L, FEV1/FVC: 65.3%). Contrast-enhanced chest CT confirmed a subpleural lesion on the mediastinal surface of the left upper lobe with an indistinct border and loss of the fat plane, suggesting direct mediastinal invasion (cT4) (Figures 1A–C). Concurrently, multiple exudative lesions were identified in both lungs, radiographically consistent with COVID-19 pneumonia (Figures 1D–F). A CT-guided percutaneous biopsy confirmed lung adenocarcinoma. Molecular profiling via amplification refractory mutation system polymerase chain reaction (ARMS-PCR) identified an EGFR exon 19 deletion. Staging workup, including brain magnetic resonance imaging and abdominal ultrasonography, showed no evidence of nodal or distant metastasis. While PET–CT was considered, the patient declined this examination due to economic considerations. Endobronchial ultrasound (EBUS) was not performed due to institutional infection control policies during the COVID-19 pandemic. The clinical stage was cIIIA (cT4N0M0) according to the 8th edition UICC/AJCC TNM staging system.

Given the concurrent diagnoses, a multidisciplinary team (MDT) was convened. In alignment with prevailing safety guidelines, surgical intervention was postponed. To mitigate the risk of oncological progression during this delay, the MDT recommended initiating bridging therapy with a third-generation EGFR-TKI. Almonertinib was selected based on its documented high efficacy and favorable safety profile, particularly its low incidence of interstitial lung disease (ILD) and minimal myelosuppression. After detailed discussion and written informed consent, neoadjuvant almonertinib (110 mg orally, once daily) was commenced.

**FIGURE 2:**
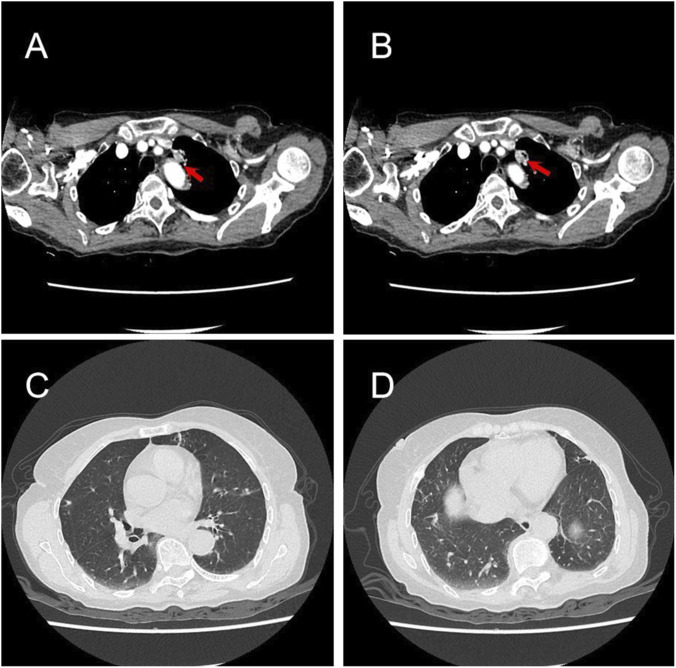
Radiographic response following 10 weeks of neoadjuvant almonertinib. **(A,B)** Post-treatment chest CT images reveal marked regression of the primary tumor (from 29 mm to 16 mm). Note the restoration of a clear fat plane between the lesion and the mediastinum (arrow). **(C,D)** Complete radiological resolution of COVID-19-related pulmonary infiltrates.

The treatment was well-tolerated without any reported adverse events. A follow-up chest CT scan after 10 weeks demonstrated pronounced regression of the primary tumor (from 29 mm to 16 mm, representing a 44.8% reduction per RECIST v1.1 criteria, consistent with partial response) and complete radiological resolution of bilateral ground-glass opacities (Figures 2A–D). Serum CEA decreased to 2.24 ng/mL. Pulmonary function tests repeated prior to surgery showed normalization (FEV1: 1.68 L, FEV1/FVC: 92.5%). Following confirmed virological clearance (negative SARS-CoV-2 PCR test) and documented radiologic tumor response, the patient underwent thoracoscopic left upper lobectomy with systematic lymph node dissection in March 2023. The surgery was uncomplicated; the tumor bed showed no significant fibrosis or adhesion attributable to neoadjuvant almonertinib, facilitating standard anatomic resection. A video recording of the procedure is provided as Supplementary Material.

**FIGURE 3:**
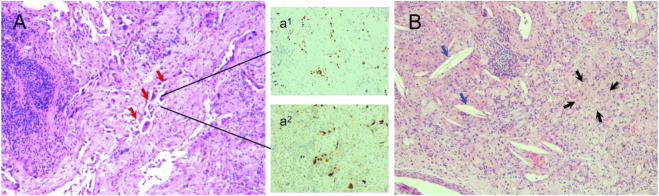
Histopathological confirmation of a major pathologic response following neoadjuvant almonertinib therapy. **(A)** Low-power view (H&E stain) showing therapy-associated fibro-inflammatory stroma with a minute focus of residual carcinoma (red arrow). The residual tumor cells show strong nuclear TTF-1 positivity (a^1^) and a low proliferative index on Ki-67 immunohistochemistry (a^2^). **(B)** Adjacent area of the tumor regression bed (H&E stain) with histiocytic aggregation (foamy macrophages, black arrows) and cholesterol clefts (blue arrows), characteristic of treatment response.

**FIGURE 4:**
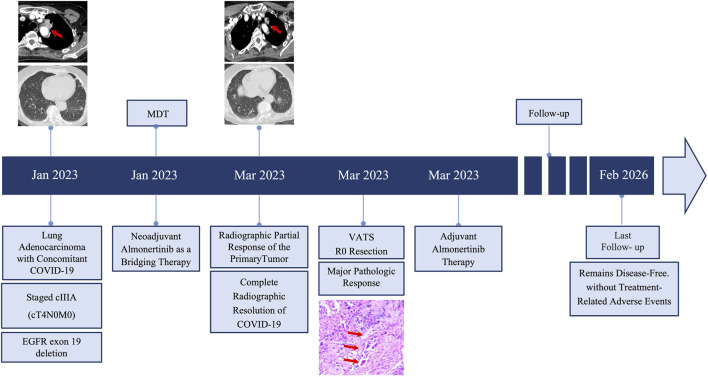
Schematic timeline of the clinical course. The diagram outlines the management sequence from initial diagnosis through neoadjuvant therapy, surgery, adjuvant therapy, and long-term follow-up.

Pathological examination confirmed a major pathologic response (MPR), defined as <5% residual viable tumor within a therapy-altered tumor regression bed featuring fibroinflammation, histiocytic aggregates, and cholesterol clefts (Figures 3A,B). All 29 dissected lymph nodes were negative for metastasis. The final pathological stage was ypIA1 (ypT1aN0). The postoperative course was uneventful. Given the profound pathological response, adjuvant almonertinib (110 mg daily) was initiated on postoperative day 2 and has been maintained with full adherence. The patient underwent surveillance at 3-month intervals, which included chest CT, brain CT/MRI, abdominal ultrasonography, and serum CEA. Over 36 months, serial imaging showed no recurrence, and serum CEA remained within normal limits. No treatment-related adverse events were observed. The patient reported full recovery of daily activities without dyspnea or fatigue, and her ECOG performance status was 0 throughout follow-up. A schematic timeline is provided in Figure 4.

## Discussion

This case report describes a novel and successful integrated strategy for managing a patient with resectable EGFR-mutant NSCLC and concomitant active COVID-19 pneumonia. While guidelines recommend delaying surgery, robust evidence on optimal bridging therapies—particularly those avoiding immunosuppression and additional pulmonary toxicity—remains scarce for this subgroup. Our approach, centered on a third-generation EGFR-TKI with a favorable safety profile, effectively bridged this high-risk period.

The management of resectable NSCLC with concomitant active COVID-19 is guided by consensus recommending surgery postponement for at least 7 weeks, which is associated with significantly reduced mortality (COVIDSurg, 2021). However, for locally advanced tumors, this delay carries a tangible risk of disease progression and potential loss of resectability. Conventional bridging strategies entail distinct risks in the context of active viral pneumonia. Platinum-based chemotherapy confers a well-documented risk of myelosuppression, which may attenuate the host antiviral immune response—a concern corroborated by the COVID-19 and Cancer Consortium (CCC19) cohort study (Kuderer et al., 2020). Conversely, ICIs are associated with immune-related pneumonitis (approximately 3%–5% in lung cancer populations) (Luo et al., 2020), and superimposing such injury upon SARS-CoV-2–induced alveolar damage could precipitate severe respiratory compromise. Consensus guidelines, therefore, recommend avoiding ICIs during active infection (Passaro et al., 2021). Stereotactic body radiotherapy (SBRT) can achieve effective local tumor control in early-stage NSCLC (Chang et al., 2015). However, in the context of active COVID-19, daily hospital visits would have substantially increased the risk of nosocomial infection. Furthermore, as a localized modality, SBRT cannot address potential micrometastatic disease. These considerations favored a systemic targeted approach.

Third-generation EGFR-TKIs appear to fulfill this theoretical requirement in principle. Established as first-line therapy for advanced EGFR-mutant NSCLC, they are increasingly demonstrating efficacy in the neoadjuvant setting. The phase II NEOS trial (CTONG 2103) of neoadjuvant osimertinib reported an objective response rate of 71% and an MPR rate of ∼25%, supporting its potential for significant tumor regression (Lv et al., 2023). Critically, their safety profile appears compatible with active COVID-19. Retrospective analyses, such as the study by Huang et al. (2024), found no evidence of increased susceptibility to SARS-CoV-2 infection or a higher risk of severe COVID-19 in patients receiving EGFR-TKIs. Real-world evidence during the pandemic further supports the feasibility of continuing these agents during active infection (Lakkunarajah et al., 2023). Almonertinib, in particular, demonstrated a low incidence of treatment-related ILD (1.4%, with grade ≥3 events in 0.2%) and no significant myelosuppression in the AENEAS trial (Lu et al., 2022). By comparison, in the landmark Phase III FLAURA trial, osimertinib was associated with an ILD incidence of 4.0% (including 1.0% grade ≥3) (Soria et al., 2018). A similar rate was reported in the ADAURA trial (3.9%) (Wu et al., 2020). Guided by this rationale, the MDT selected almonertinib as bridging therapy. The treatment was well-tolerated, and after two cycles, the patient recovered from COVID-19 and achieved marked tumor regression, enabling curative surgery. The safety profile was consistent with historical data from the AENEAS trial, with no interstitial lung disease, myelosuppression, or hepatotoxicity observed. Although concerns such as drug–infection interactions or delayed viral clearance may arise in the setting of active COVID-19, these were not encountered in this case.

The achievement of MPR is a pivotal finding. MPR (≤10% viable tumor) following neoadjuvant therapy is a validated surrogate endpoint associated with improved long-term survival in resectable NSCLC (Hellmann et al., 2014). The observation of less than 5% residual viable tumor cells, together with a partial response per RECIST criteria (44.8% reduction), underscores the robust antitumor activity of neoadjuvant almonertinib in this case. This exceptional response justified continuing the same targeted agent in the adjuvant phase, supported by the ADAURA trial, which established the survival benefit of adjuvant osimertinib after complete resection (Wu et al., 2020). Thus, our strategy established a coherent precision oncology pathway from preoperative bridge through adjuvant consolidation.

The success of this integrated approach is reflected in multiple favorable outcomes: complete resolution of COVID-19 pneumonia enabling safe surgery, successful R0 resection with MPR, and sustained DFS for 33 months with excellent tolerability. However, several limitations must be acknowledged. First, as a single-case report, generalizability is limited; efficacy and safety require validation in larger prospective studies. Second, the optimal duration of neoadjuvant TKI bridging remains undefined and may vary depending on tumor biology and the course of COVID-19. Third, despite prolonged efficacy, long-term surveillance for acquired resistance remains imperative. Future research should aim to identify biomarkers predictive of pathological response and define the role of this approach across tumor stages and COVID-19 severities.

## Conclusion

In conclusion, this case demonstrates that a precision bridging strategy with neoadjuvant almonertinib represents a potentially viable approach that warrants further investigation for patients with resectable EGFR-mutant NSCLC and active COVID-19 pneumonia. By offering targeted antitumor activity without compounding the risks of immunosuppression or significant pulmonary toxicity, this approach presents a clinically reasoned solution to a complex dilemma, allowing for concurrent management of both life-threatening conditions. It underscores the potential of targeted agents in tailored bridging therapies and highlights the need for further study in this area.

## Data Availability

The original contributions presented in the study are included in the article/Supplementary Material; further inquiries can be directed to the corresponding authors.
